# *COL5A1* gene variants previously associated with reduced soft tissue injury risk are associated with elite athlete status in rugby

**DOI:** 10.1186/s12864-017-4187-3

**Published:** 2017-11-14

**Authors:** Shane M. Heffernan, Liam P. Kilduff, Robert M. Erskine, Stephen H. Day, Georgina K. Stebbings, Christian J. Cook, Stuart M. Raleigh, Mark A. Bennett, Guan Wang, Malcolm Collins, Yannis P. Pitsiladis, Alun G. Williams

**Affiliations:** 1 MMU Sports Genomics Laboratory, Manchester Metropolitan University, Crewe, Manchester UK; 2 A-STEM, College of Engineering, Swansea University, Swansea, UK; 3 Research Institute for Sport & Exercise Sciences, Liverpool John Moores University, Liverpool, UK; 4 Institute of Sport, Exercise and Health, University College London, London, UK; 5 School of Sport, Health and Exercise Sciences, Bangor University, Bangor, UK; 6 Centre for Physical Activity and Chronic Disease, Institute of Health and Wellbeing, University of Northampton, Northampton, UK; 7 FIMS Reference Collaborating Centre of Sports Medicine for Anti-Doping Research, University of Brighton, Brighton, UK; 8 Division of Exercise Science and Sports Medicine, Department of Human Biology, University of Cape Town (UCT), Cape Town, South Africa; 9 School of Public Health, Physiotherapy and Sports Science, University College Dublin, Dublin 4, Ireland

**Keywords:** Rugby union, Rugby league, Tendon, Ligament, Genetics

## Abstract

**Background:**

Two common single nucleotide polymorphisms within the *COL5A1* gene (SNPs; rs12722 C/T and rs3196378 C/A) have previously been associated with tendon and ligament pathologies. Given the high incidence of tendon and ligament injuries in elite rugby athletes, we hypothesised that both SNPs would be associated with career success.

**Results:**

In 1105 participants (RugbyGene project), comprising 460 elite rugby union (RU), 88 elite rugby league athletes and 565 non-athlete controls, DNA was collected and genotyped for the *COL5A1* rs12722 and rs3196378 variants using real-time PCR. For rs12722, the injury-protective CC genotype and C allele were more common in all athletes (21% and 47%, respectively) and RU athletes (22% and 48%) than in controls (16% and 41%, *P* ≤ 0.01). For rs3196378, the CC genotype and C allele were overrepresented in all athletes (23% and 48%) and RU athletes (24% and 49%) compared with controls (16% and 41%, *P* ≤ 0.02). The CC genotype in particular was overrepresented in the back and centres (24%) compared with controls, with more than twice the odds (OR = 2.25, *P* = 0.006) of possessing the injury-protective CC genotype. Furthermore, when considering both SNPs simultaneously, the CC–CC SNP-SNP combination and C–C inferred allele combination were higher in all the athlete groups (≥18% and ≥43%) compared with controls (13% and 40%; *P* = 0.01). However, no genotype differences were identified for either SNP when RU playing positions were compared directly with each other.

**Conclusion:**

It appears that the C alleles, CC genotypes and resulting combinations of both rs12722 and rs3196378 are beneficial for rugby athletes to achieve elite status and carriage of these variants may impart an inherited resistance against soft tissue injury, despite exposure to the high-risk environment of elite rugby. These data have implications for the management of inter-individual differences in injury risk amongst elite athletes.

## Background

Elite rugby athletes regularly experience high velocity collisions that lead to increasingly high injury occurrence rates that are likely to be a consequence of the increasing size and strength of the athletes [1–4]. This increased size and strength is likely to result in greater changes in momentum during player collisions, as well as during voluntary accelerations and decelerations. This has resulted in rugby union (RU) having one of the highest reported injury incidence rates in professional team sports [5]. Meta-analyses have shown that for every 1000 h, an elite RU athlete will experience approximately 81 injuries during match play and three during training, with the majority being ligament, tendon and muscle injuries of the lower limbs [6]. Indeed, in the most recent Rugby World Cup (2015) this rate of incidence was more than 90 injuries per 1000 h [7]. Furthermore, pooled data from 10 studies of elite rugby league (RL) athletes show that injury incidence is approximately twice (172 per 1000 h) that of RU [8]. Similar to RU, the majority of injuries in RL occur on the lower limbs, consisting mainly of sprains and strains [8]. Injury incidence differs across RU playing position, with elite back row players showing the highest rate among forwards and centres the highest among backs [7]. Therefore, investigating the molecular genetic components of these injuries, including in the context of playing positions that differ in terms of physiological characteristics [9, 10], match play demands [11] as well as genetically [12], may progress understanding towards greater individualisation of match play exposure and training load and mode, in order to reduce injury risk [13].

The collagen fibril, which consists predominately of type I collagen, is the primary structural component of tendons, ligaments and other non-cartilaginous connective tissues [14]. The formation and diameter of the collagen fibril is regulated by, amongst other molecules, the minor fibrillar type V collagen protein [15–18]. The type V collagen isoform comprises two α1(V) and one α2(V) chains, encoded by the *COL5A1* and *COL5A2* genes respectively [16, 19] and forms between 1 and 5% of total collagen content [18, 20]. The *COL5A1* gene is the most explored genetic locus in relation to tendon and ligament injuries [21–25], while mutations in the *COL5A1* gene have been identified in Ehlers-Danlos syndrome, a disease characterised by joint hypermobility, laxity and muscle hypotonia [26]. This results in irregularly large collagen fibrils within connective tissue [27] and is attributed to a reduced synthesis of collagen type V [17, 28].

Two common single nucleotide polymorphisms (SNPs, rs12722 C/T and rs3196378 C/A) located in the functional 3′ untranslated region (3′ UTR) *COL5A1* gene on chromosome 9 have been associated with tendon [23] and ligament ([rs12722] [29]) injuries. Specifically, the CC genotype of the more extensively investigated rs12722 polymorphism has been previously associated with reduced risk of chronic Achilles tendinopathy (odds ratio (OR) = 0.42-AUS and OR = 0.38-SA, respectively; [23, 25]), anterior cruciate ligament injury in females (OR = 6.6; [22]) and lateral epicondylitis (OR = 1.4; [21]), suggesting a protective role of the C allele against injury. Although there are conflicting results [23, 30], our current understanding also suggests that the CC genotype and/or the C allele of rs3196378 would also have a protective role [31, 32]. Considering the high frequencies of tendon and ligament inquiries in elite rugby [6–8, 33], assessing these specific genetic variants may be of use in helping improve the management of injury risk in individual players.

Given the association of the two *COL5A1* gene variants with injury risk, it is possible that possession of the risk alleles might reduce an individual’s ability to withstand exposure to the environment of competitive rugby without suffering more frequent injuries. Consequently, those individuals would be forced to miss training, selection and competitive events important for their career progression. Thus, athletes carrying the C allele at either or both rs12722 and rs3196378 might be at an advantage in terms of their ability to achieve success in elite competitive rugby and at a disadvantage in terms of their shorter-term and longer-term musculoskeletal health. Therefore, the objective of the present study was to investigate if *COL5A1* rs12722 and rs3196378 genotype and allele frequencies differed between elite rugby athletes and a control population, and/or between playing positions. It was hypothesised that the *COL5A1* rs12722 and rs3196378 injury-protective C alleles and/or CC genotypes would be overrepresented in elite rugby athletes compared with controls.

## Methods

### Participants

As part of the ongoing RugbyGene project [12, 13, 34], a total of 1105 individuals were recruited and gave written informed consent to participate in the present study. An a priori calculation for 80% power to detect a small effect size (w) of 0.1 required a sample of >785 participants. The sample comprised elite Caucasian male rugby athletes (*n* = 540; mean (standard deviation) height 1.85 (0.07) m, mass 101 (14) kg, age 29 (7) years) including 72% British, 16% South African, 7% Irish and 5% of other nationalities. Caucasian controls (68% male; *n* = 565; height 1.75 (0.10) m, mass 75 (13) kg, age 26 (11) years) included 86% British, 12% South African, 1% Irish and 1% of other nationalities recruited mainly during 2012–2016. Eight athletes competed in both elite RL and RU and were included in both groups that were analysed separately. Athletes were considered elite if they had competed regularly (> 5 matches) since 1995 in the highest professional league in the UK, Ireland or South Africa for RU and the highest professional league in the UK for RL. Of the RU athletes, 51.7% had competed at international level for a “High Performance Union” (Regulation 16, worldrugby.org) and 43.2% of RL had competed at international level. All data for the athlete group’s international status were confirmed as of 1st January 2017. Most participants in the current study were also participants in previous publications regarding variations in the *ACTN3*, *ACE* and *FTO* genes [12, 34].

### Sample collection and genotyping

Description of all molecular procedures have previously been described in detail [12]. Briefly, blood (~70% of all samples), saliva (~25%) or buccal swab samples (~5%) were obtained via the following protocols. Blood from a superficial forearm vein was put into an EDTA tube and stored in sterile tubes at −20 **°**C until processing. Saliva samples were collected using Oragene DNA OG-500 collection tubes (DNA Genotek Inc., Ontario, Canada) according to the manufacturer’s protocol and stored at room temperature until processing. Sterile buccal swabs (Omni swab, Whatman, Springfield Mill, UK) were rubbed against the buccal mucosa of the cheek for approximately 30 s and the tips stored at −20 **°**C until processing. At MMU and Glasgow, DNA was stored at 4 **°**C following isolation performed using the QIAamp DNA Blood Mini kit and standard spin column protocol (Qiagen, West Sussex, UK). In Cape Town, DNA was isolated from whole blood [35] and samples stored at −20 **°**C. At Northampton, DNA was isolated from whole blood using Flexigene kits (Qiagen), with the resulting samples stored at −20 **°**C.

Genotyping at all three centres was performed using TaqMan assays (Applied Biosystems, Paisley, UK) for both the *COL5A1* rs12722 and rs3196378 variants. Our genotyping methods and quality control procedures have been fully described in our earlier study [12]. Minor adaptations were made to the volumes used in each assay mix depending on whether the DNA was obtained from buccal swabs or saliva/blood. PCR was performed on either a Chromo4 (Bio-Rad, Hertfordshire, UK) or StepOnePlus thermal cycler (Applied Biosystems). Genotypes were called based on reporter dye intensity and visualized using cluster plots. The TaqMan assays included VIC and FAM dyes that for rs12722 indicated C and T alleles on the forward DNA strand, respectively. Thus, VIC/FAM were interpreted as: 5′-CACACCCA[C/T]GCGCCCCG-3′. For rs3196378, VIC and FAM dyes indicated C and A alleles on the forward DNA strand, respectively and were interpreted as: 5′-CCCACCCC[A/C]GCCCTGGC-3′. Genotype calling was 100% successful for both polymorphisms in the athlete samples. For rs12722, one of the 566 control samples was unsuccessful and, for rs3196378, 10 of the 566 control samples were unsuccessful. There was 100% agreement among reference samples genotyped in the three genotyping centres, i.e. Glasgow, Northampton and MMU laboratories.

### Data analysis

SPSS for Windows version 22 (SPSS Inc., Chicago, IL) software was used to conduct Pearson’s Chi-square (χ^2^) tests to compare genotype (using three analysis models; additive, recessive and dominant), allele and inferred haplotype frequencies between athletes and controls, and between RU subgroups based on playing position and controls. With 80% statistical power, analyses of all genetic models in positional subgroups compared with controls (forwards, backs and back 3-centres) were able to detect a small-to-medium effect size (w) of 0.12. Multifactor Dimensionality Reduction (MDR; www.multifactordimensionalityreduction.org) software was used to calculate SNP-SNP epistasis interactions [36]. Haplotypes were inferred using SNPStats [37]. Sixty five tests were subjected to Benjamini-Hochberg (BH; [38]) corrections to control false discovery rate and corrected probability values are reported. Odds ratios (OR) were calculated to estimate effect size. CubeX online software (www.oege.org/software/cubex) was used to determine linkage disequilibrium statistics [39]. Alpha was set at 0.05.

## Results

Genotype frequencies were in Hardy-Weinberg equilibrium for both rs12722 and rs3196378 in the control (*P* ≥ 0.21) and athlete groups (RL, *P* ≥ 0.11; RU, *P* ≥ 0.754). There was no sexual dimorphism of genotype frequency for either rs12722 (*P* = 0.279) or rs3196378 (*P* = 0.374) within the control group. *COL5A1* rs12722 and rs3196378 were in tight linkage disequilibrium for both controls (D′ = 0.902, r^2^ = 0.784) and all athletes (D′ = 0.877, r^2^ = 0.738). Athletes were taller and heavier (*P* < 0.05) but not older (*P* > 0.05) than controls.

### rs12722

**Fig. 1:**
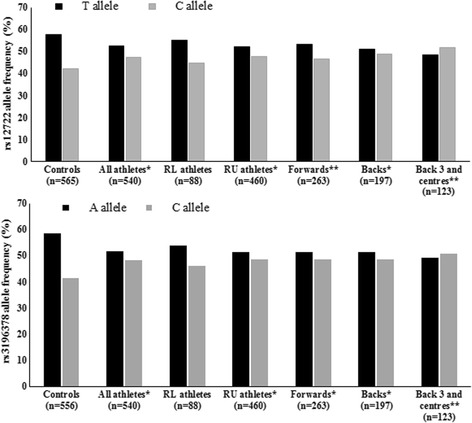
Allele frequency of *COL5A1* rs12722 ( **a** ) and rs3196378 ( **b** ) for control and athlete groups. Asterisks indicate a difference in allele frequency between the particular athlete group and controls. A single asterisk (*) designates *P* = 0.01 and a double asterisk (**) designates *P* = 0.02. RU, rugby union, RL rugby league. Eight athletes competed in both elite RL and RU and were included in both groups that were analysed separately

The CC genotype, proportion of C allele carriers and C allele were overrepresented in all athletes (21.1%, 73.3% and 47.2%, respectively) and RU athletes (22.0%, 73.3% and 47.6%) compared with controls (15.6%, 66.5% and 41.1%, Table 1 and Fig. 1, *P* ≤ 0.01). Furthermore, the CC genotype, proportion of C allele carriers (Table 1) and C allele (Fig. 1) were overrepresented in the subgroups of RU forwards (22.1%, 71.5% and 46.8%) and backs (21.8%, 75.6% and 48.7%) compared with controls (15.6%, 66.5% and 41.1%). Additionally, of the RU subgroups, the back three and centres differed from controls and showed the greatest C allele and CC genotype frequency (50.8% versus 41.1% and 24.4% versus 15.6%, respectively, Table 1 and Fig. 1, *P* ≤ 0.02). Compared with controls, those back three and centre players had 2.3 times the odds of possessing the CC genotype and 1.5 times the odds of possessing the C allele (Table 2).

**Table 1:** *COL5A1* rs12722 and rs3196378 genotype and allele distribution of controls and athletes separated by code (RL and RU) and into positional sub-groups for RU, presented as genotype/allele counts followed by percentage in parentheses

Genotype	Controls	All athletes	RL athletes	RU athletes	Forwards	Backs	Back 3 and Centres
*rs12722*							
TT	189 (33.4)	144 (26.7)	23 (26.1)	123 (26.7)	75 (28.5)	48 (24.4)	28 (22.8)
CT	288 (51.0)	282 (52.2)	51 (58.0)	236 (51.3)	130 (49.4)	106 (53.8)	65 (52.8)
CC	88 (15.6)	111 (21.1)*	14 (15.9)	101 (22.0)*	58 (22.1)*	43 (21.8)*	30 (24.4)*
Total	565	540	88	460	263	197	123
T allele carriers	477 (84.4)	426 (78.9)*	74 (84.1)	359 (78.0)*	205 (77.9)*	154 (78.2)*	93 (75.6)*
C allele carriers	379 (66.5)	396 (73.3)*	65 (73.9)	337 (73.3)*	188 (71.5)	149 (75.6)*	95 (77.2)*
*rs3196378*							
AA	183 (32.9)	144 (26.7)	23 (26.1)	123 (26.7)	74 (28.1)	49 (24.9)	28 (22.8)
CA	286 (51.4)	271 (50.2)	49 (55.7)	227 (49.3)	122 (46.4)	105 (53.3)	65 (52.8)
CC	87 (15.6)	125 (23.1)*	16 (18.2)	110 (24.0)*	67 (25.5)*	43 (21.8) *	30 (24.4)*
Total	556	540	88	460	263	197	123
A allele carriers	469 (84.4)	415 (76.9)*	72 (81.8)	350 (76.1)*	196 (74.5)*	154 (78.2)*	93 (75.6)*
C allele carriers	373 (67.1)	396 (73.3)*	65 (73.9)	337 (73.3)*	189 (71.9)	148 (75.1)*	95 (77.2)*

RU, rugby union, RL rugby league. The genotype, T allele carrier and C allele carrier data represent the additive, dominant model and recessive models, respectively. Eight athletes competed in both elite RL and RU and were included in both groups that were analysed separately

Asterisks (*) indicate Chi-square differences from controls (*P* ≤ 0.03)

**Table 2:** Odds ratio statistics for RU player status of *COL5A1* gene variants (rs12722 and rs3196378)

Positional comparison	Model	^b^Odds Ratio	95% Confidence Interval
rs12722			
All athletes v Controls	CC/TT CC/T_carriers_ C_allele_/T_allele_	1.70 1.45 1.28	1.19–2.42 1.07–1.97 1.08–1.52
RU athletes v Controls	CC/TT CC/T_carriers_ C_allele_/T_allele_	1.76 1.53 1.30	1.22–2.54 1.11–2.09 1.09–1.55
Forwards v Controls	CC/TT CC/T_carriers_ C_allele_/T_allele_	1.66 1.53 1.26	1.08–2.54 1.06–2.22 1.02–1.55
Backs v Controls	CC/TT CC/T_carriers_ C_allele_/T_allele_	1.92 1.51 1.36	1.19–3.12 1.01–2.27 1.08–1.72
Back 3 and centres v Controls	CC/TT CC/T_carriers_ C_allele_/T_allele_	2.30 1.76 1.48	1.29–4.08 1.09–2.78 1.12–1.96
rs3196378			
All athletes v Controls	CC/AA CC/A_carriers_ C_allele_/A_allele_	1.83 1.66 1.32	1.29–2.59 1.22–2.25 1.12–1.56
RU athletes v Controls	CC/AA CC/A_carriers_ C_allele_/A_allele_	1.88 1.73 1.34	1.31–2.70 1.27–2.37 1.12–1.59
Forwards v Controls	CC/AA CC/A_carriers_ C_allele_/A_allele_	1.90 1.88 1.34	1.25–2.89 1.31–2.69 1.09–1.66
Backs v Controls	CC/AA CC/A_carriers_ C_allele_/A_allele_	1.85 1.54 1.33	1.14–2.99 1.02–2.31 1.06–1.68
Back 3 and centres v Controls	CC/AA CC/A_carriers_ C_allele_/A_allele_	2.25 1.78 1.46	1.27–4.00 1.11–2.84 1.11–1.93
Haplotypes and SNP epistasis			
All athletes v Controls	C–C/T–A ^a^CC–CC	1.31 1.97	1.10–1.56 1.44–2.69
RU athletes v Controls	C–C/T–A ^a^CC–CC	1.32 2.11	1.11–1.59 1.53–2.91
Forwards v Controls	C–C/T–A ^a^CC–CC	1.31 2.19	1.05–1.62 1.51–3.16
Backs v Controls	C–C/T–A ^a^CC–CC	1.36 1.86	1.07–1.72 1.29–2.67
Back 3 and centres v Controls	C–C/T–A ^a^CC–CC	1.49 2.35	1.12–1.99 1.47–3.77

^a^MDR best model versus all other genotype combinations for rs12722 and rs3196378

^b^All odds ratios were statistically significant (*P* < 0.05)

### rs3196378

The CC genotype, proportion of C allele carriers and C allele were overrepresented in all athletes (23.1%, 73.3% and 48.4%) and RU athletes (23.9%, 73.3% and 48.6%) compared with controls (15.6%, 67.7% and 41.4%, Table 1 and Fig. 1, *P* ≤ 0.02). Furthermore, CC genotype, proportion of C allele carriers (Table 1) and C allele (Fig. 1) were overrepresented in backs (21.8%, 75.1% and 48.5%) compared with controls (15.6%, 67.7% and 41.4%, *P* ≤ 0.02). Forwards also had higher CC genotype and C allele frequencies (25.5% and 48.7%; Table 1 and Fig. 1) and showed almost twice the odds of being CC genotype than carrying an A allele, compared with controls (Table 2). For the back three and centres group, 24.4% were CC genotype, 77.2% were C allele carriers and C allele frequency was 47.8% - all of which were greater than controls (*P* ≤ 0.02; Table 1 and Fig. 1, OR = 2.25, Table 2).

### Haplotype and SNP epistasis analysis

**Fig. 2:**
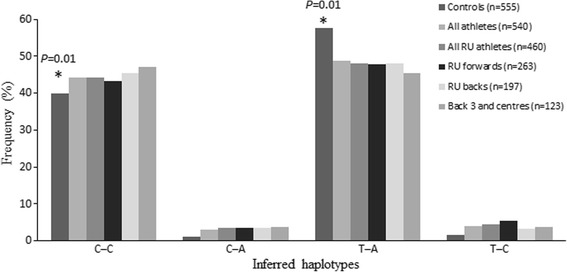
Inferred haplotype frequencies derived from *COL5A1* rs12722 and rs3196378. RU, rugby union. Asterisks (*) indicate differences in inferred haplotype frequencies between the controls and each athlete group ( *P* = 0.01). Eight athletes competed in both elite RL and RU and were included in both groups that were analysed separately

There was a greater frequency of the CC–CC SNP-SNP combination in all athletes (18.3%), RU athletes (18.9%), RU forwards (19.4%), RU backs (18.3%) and to the greatest extent the back three and centre group (20.3%; OR = 2.35; Table 2), compared with control (12.8%; all athlete comparisons with the control group were *P* = 0.01). Furthermore, C–C inferred haplotype frequencies were higher in all the athlete groups compared with controls, reflected by a greater frequency of the T–A inferred haplotype in the control group (*P* = 0.01; Fig. 2).

## Discussion

The present observations are the first to identify associations between *COL5A1* rs12722 and rs3196378 polymorphisms and athlete status in a large cohort of elite rugby athletes. As hypothesised, the apparent injury-protective C allele and CC genotype, of both SNPs [23], were overrepresented in elite rugby athletes compared with controls. This association persisted across playing position, with the C allele being overrepresented in RU forwards and backs including the back three and centres group, compared with controls. Furthermore, when the two SNPs are combined, the CC–CC combination and C–C inferred allele combination showed similar overrepresentation in elite rugby athletes compared with controls.

The results provide an insight into the potential injury susceptibility of some elite rugby athletes. September et al. [23] identified a higher frequency of the CC genotype in asymptomatic controls for both rs12722 and rs3196378 compared with tendinopathy patients [23, 32]. Moreover, the rs12722 T allele has been associated with ligament injury [21, 22, 24, 40] and Achilles tendinopathy [23, 25] with the C allele again identified as protective in these studies, despite a lack of replication in another study [30]. Greater joint laxity has almost a 3-fold increase in risk of knee ligament rupture [41] and greater joint laxity has recently been associated with the rs12722 T allele in non-white females [40]. Collectively, these data suggest that the C allele of rs12722 and/or rs3196378 (or other variant(s) in strong LD with these SNPs) might be beneficial in protecting against tendon and ligament injuries. This is reflected in the present study showing greater C allele frequency in elite rugby athletes compared with controls. Based on these data, we propose that when exposed to the high-risk environment of rugby during training and especially during competitive matches, ceteris paribus, carriage of the C alleles at the rs12722 and rs3196378 loci provides both a shorter-term and longer-term advantage to rugby athletes in the form of reduced injury risk. Athletes with fewer and/or less severe injuries, all else being equal, will miss fewer matches, training and selection events and thus be more likely to progress towards elite status in their athletic careers compared with their peers.

The rs12722 CC genotype has also been related to a lower incidence of exercise-associated muscle cramping (EAMC) in Caucasian ironman and ultra-marathon athletes [42]. The authors hypothesised that this was due to similar mechanisms of reduced tendon injury susceptibility, in that rs12722 alters soft tissue structural and mechanical properties (tissue stiffness and thickening). Some recent findings might support this hypothesis, as greater tendon stiffness was associated with the rs12722 T allele in one study [43], however another study reported no association of rs12722 with tendon structural or mechanical properties [44]. These data suggest that in addition to the apparent protection from tendon and ligament injury, the greater frequencies of the C allele in elite rugby athletes might be protective against muscle cramping and possibly reduced tendon stiffness. Indeed, recent evidence from elite RL shows that over 70% of athletes experience EAMC per season and that history of cramping is the strongest predictor of future EAMC [45]. In contrast, the TT genotype has been associated with greater endurance running ability of Caucasian ironman triathletes (TT = 294 min, CC = 307 min; [46]). However, recent data show no association of rs12722 with either running economy or VȮ_2max_ [47]. While endurance capacity is of value in elite rugby, the predominant focus of player selection and training programs is towards power, speed and strength - i.e. short-term, anaerobic performance [with notable differences between playing positions; 9, 10].

Limited data exist regarding *COL5A1* genetic variation and team sport athletes. In a study of 73 soccer athletes, including some elite players, no rs12722 TT genotype individuals were identified (a potentially interesting observation but difficult to interpret because of the varied geographic ancestry of the athletes), but there was a tendency for more severe muscle injuries in the TC genotype group (*P* = 0.08), compared with CC [48]. Here, consistent with those observations, we show an overrepresentation of the protective C allele and CC genotype of both rs12722 and rs3196378, in addition to CC–CC SNP-SNP combination and C–C inferred allele combination in elite rugby athletes.

Some possible mechanisms have been proposed to explain the association of *COL5A1* gene variants and soft tissue injury [31, 32]. Laguett et al. [32] have shown that the *COL5A1* 3′ UTR – where both rs12722 and rs3196378 are situated - affects mRNA stability. For both SNPs, the alleles associated with greater soft-tissue injury risk were associated with greater Hsa-miR-608 stability, which in turn may alter the Col5α1 protein secondary structure - proposed to play a role in type V collagen production [31]. This would suggest that C/T allele differences at rs12722 might alter the co-polymerisation of collagen type V and type I fibrils. However, to date, this has not been demonstrated experimentally and exactly how this may translate into functional properties is currently unknown. Nevertheless, it appears that the C allele and CC genotype of rs12722 and rs3196378 appear beneficial for rugby athletes to achieve elite status, probably through greater resistance to soft tissue injury. Interestingly, while most relevant investigations have focussed on rs12722, we show in a large cohort (total *n* = 1090) that strong linkage disequilibrium exists in both controls and athletes between rs12722 and rs3196378. As such, it is likely that the associations of rs12722 with tendon and ligament injuries would be similar for rs3196378. It is possible that combining genetic data from multiple gene variants associated with injury susceptibility, such as those presented here, with other indicators of injury risk and recovery during rehabilitation could be used to better manage the prevention and recovery from elite player injury in the future.

## Conclusion

In conclusion, we have presented the first associations between *COL5A1* 3′ UTR rs12722 and rs3196378 and elite status within a large cohort of rugby athletes. The C alleles of both polymorphisms, separately and in combination, were overrepresented in all athletes, RU forwards, backs and in RU back three and centre players versus controls. We propose that rugby athletes possessing more C alleles at these two genetic loci are probably at a lower risk of injury, given their exposure to the high-risk environment of elite rugby. However, these data pertain to only two SNPs of many that may be relevant to soft tissue injury and interpretation of the present results should be in that context. Future investigations should seek to combine elite rugby genotype data such as these with injury incidence data during rugby matches and training. It will be important to establish whether inter-individual differences in injury risk, within a population that we demonstrate here appear to be at an overall lower genetic risk of tendon and ligament injury compared with non-athletes, nevertheless are associated with those same genetic loci.

## Abbreviations

3′ UTR: 3′ untranslated region
*COL5A1*: collagen type V α1
*COL5A2*: collagen type V α2
DNA: deoxyribonucleic acid
EAMC: exercise-associated muscle cramping
EDTA: ethylenediaminetetraacetic acid
MDR: multifactor dimensionality reduction
miRNA: micro ribonucleic acid
PCR: Polymerase chain reaction
RL: rugby league
RU: rugby union
SNP: single nucleotide polymorphisms
